# Controversies in bipolar disorder; role of second-generation antipsychotic for maintenance therapy

**DOI:** 10.1186/s40345-019-0145-0

**Published:** 2019-03-27

**Authors:** Sameer Jauhar, Allan H. Young

**Affiliations:** 1 Department of Psychological Medicine, Institute of Psychiatry, Psychology and Neuroscience, King’s College, London, SE5 8AF UK; 2 Psychosis Clinical Academic Group, South London and Maudsley NHS Foundation Trust, London, UK

## Abstract

In this narrative review, we discuss use of second-generation antipsychotics (SGAs) in maintenance treatment of bipolar disorder. We compare their use to historically more established treatments (particularly lithium, the gold standard). To compare we review evidence on efficacy, effectiveness and tolerability across illness poles, possible mechanisms of treatment response, guidance given by guideline groups and pragmatic clinical considerations. We then illustrate the controversies in maintenance antipsychotic use, with the example of first episode mania and its treatment within first episode psychosis services. Finally, we make suggestions for future studies to unpick these differences.

## Introduction

Bipolar disorder (BD), as defined by modern classification systems, encompasses episode of mania/hypomania and usually depression. Although acute episodes are significant, it is the recurrent nature of the illness that has implications for longer-term functioning and places it as the 4th leading cause of disability adjusted life years in people aged 10–24 (Gore et al. 2011).

A careful review on illness course in the pre-medication and post-medication era suggests recurrence to be very much the rule, as opposed to the exception (Goodwin and Jamison 2007). High rates of recurrence are seen uniformly throughout illness course, from first episode onwards (Gignac et al. 2015). Because of the high risk of episode recurrence, prophylactic treatment is usually an essential part of treatment. Given that a significantly higher proportion of people experience periods of depression than (hypo)mania (Judd et al. 2002) treatments should be examined for efficacy for this pole of the illness in addition to mania.

Current drugs approved for maintenance therapy in bipolar disorder by the United States (US) Food and Drugs Administration (FDA) are; lithium, lamotrigine, aripiprazole, olanzapine, quetiapine (immediate-release) and ziprasidone. Approval has also been given to risperidone long acting injection as monotherapy or adjunct, as well as aripiprazole long acting injection for monotherapy in Bipolar 1 disorder (Vieta et al. 2018).

## Evidence for the gold standard-lithium

Lithium has been a cornerstone of maintenance treatment in BD since the 1960s, the first report of prophylactic effects that we are aware of being a report by Hartigan in (1963). Initial methodological problems (e.g., discontinuation effects causing rebound mania (Suppes et al. 1991) were addressed by subsequent trials, in which lithium was used as comparator to placebo, and in latter studies, antipsychotics. Meta-analyses versus placebo, from 7 trials, indicated benefits of lithium in preventing all mood episodes (RR = 0.66), manic episodes (RR = 0.52) and to a lesser extent, depressive episodes (RR = 0.78). When compared to anticonvulsants, lithium demonstrated superiority in preventing manic episodes (RR = 0.66), though not depressive episodes. The longest trial length was 2 years, and all but one trial included people with Bipolar 1 disorder (Severus et al. 2014). Lithium has also been suggested to have anti-suicide effects (Lewitzka et al. 2015). Effects on cognitive function are heterogenous, with small impairments in verbal learning and memory, though no effects on domains such as executive function or processing speed, with a suggestion of worsening in psychomotor speed over time (Wingo et al. 2009).

The risks of lithium are clearly delineated and include side-effects of treatment (Shine et al. 2015) and rebound mania upon discontinuation (Suppes et al. 1991). In the former review, the authors examined laboratory data in people taking lithium, versus controls finding increased risk for stage 3 kidney disease (HR = 1.93), hypothyroidism (HR = 2.31), raised calcium (HR = 1.43). Real-world data on lithium discontinuation taken from an epidemiologically defined area over a 48-year period, of people taking lithium, suggested 54% stopped it completely. Of the 561 episodes of discontinuation, the majority of people stopped due to adverse events (62%), the most prevalent being renal disease, diarrhoea and tremor. Psychiatric reasons caused discontinuation in 44% of people, the most common being non-adherence and perceived lack of effectiveness (Öhlund et al. 2018).

## Evidence for second-generation antipsychotics-how do they compare?

The use of antipsychotics in BD dates back to the 1960s, predominantly in the acute phase, compared to placebo (Klein and Oaks 1967) and lithium, in acute mania (Prien et al. 1972), as well as functional psychosis (Johnstone et al. 1988). The Prien et al. trial found comparable efficacy to lithium in acute mania, and the latter significant effects of pimozide, though not lithium, on psychotic symptoms, in undifferentiated psychosis. Antipsychotic efficacy in acute mania is clear, meta-analytic evidence suggesting greater efficacy for haloperidol than lithium (SMD 0.19), with heterogeneity between compounds, with predominantly dopamine antagonists such as haloperidol having greatest efficacy (Cipriani et al. 2011).

The earliest observational study of antipsychotics for maintenance treatment appears to be the use of clozapine in people attending a service for resistant mood disorders (Zarate et al. 1995). An advantage of the second-generation antipsychotics (SGAs) was their decreased propensity to cause movement disorder, in comparison to first-generation antipsychotics (FGAs), a 2017 meta-analysis showing a statistically significant difference in tardive dyskinesia between classes (around 20% compared to 30%), with lower prevalence in FGA naïve subjects (around 7%) (Carbon et al. 2017). Other side-effects, such as metabolic effects are more prevalent with SGAs (see below). A 2017 systematic review and meta-analysis of SGAs in maintenance treatment of BD included 15 RCTs, lasting from 6 months to 2 years, and one observational study lasting 4 years (Lindström et al. 2017). This examined monotherapy and adjunctive therapy to lithium, sodium valproate or lamotrigine. Antipsychotics examined included olanzapine (4 trials), quetiapine (4 trials), aripiprazole (3 trials), risperidone (3 trials) and ziprasidone (1 trial). Meta-analyses showed antipsychotic monotherapy to be superior to placebo for reducing overall relapse risk (olanzapine: RR 0.52 (95% CI 0.38–0.71), 2 studies; quetiapine: HR 0.37 95% CI 0.31–0.45), 2 studies; risperidone: RR 0.61 (95% CI 0.47–0.80), 2 studies), though the quality of studies, using GRADE, was very poor. As an adjunct to conventional mood stabilizers (lithium/valproate/lamotrigine), when given to people who had responded to acute treatment, there was a beneficial effect for aripiprazole (RR 0.65, 95% CI 0.50–0.85; 2 studies), olanzapine (RR = 0.49 (95% CI 0.27–0.91; one study), quetiapine (RR 0.38, 95% CI 0.32–0.46; 2 studies) and ziprasidone (RR 0.62, 95% CI 0.40–0.96; 1 study). One trial with risperidone long-acting injection (LAI) in people with Bipolar 1 disorder and 4 or more episode in the prior year, was not statistically significant in the meta-analysis for relapse to mania or depression, though did show benefit in a 52 week follow-up compared to placebo as an add-on to treatment as usual, with a 2.3 fold decreased risk of relapse to any mood episode (Macfadden et al. 2009). Adjunctive treatment with quetiapine was the only drug that reduced both manic (RR 0.39, 95% CI 0.30–0.52; 2 studies) and depressive (RR 0.38, 95% CI 0.29–0.49; 2 studies) episodes. All but one study had an enriched design, i.e., patients were taking the drug prior to randomization, essentially a form of selection bias. Two of the RCTs included people with Bipolar 2 disorder (Table 1).

**Table 1:** Polarity, receptor affinities and side-effects of antipsychotics used in the maintenance treatment of bipolar disorder

Antipsychotic	Polarity^a^	Receptor profile in order of affinity^b^	Common side effects^b^
Aripiprazole	Mania, mixed features	D_2_ partial agonist 5HT_2A_ antagonist 5HT_1A_ partial agonist 5HT_7_ antagonist 5HT_2C_ partial agonist α_2_ antagonist	Akathisia, headache, nausea
Asenapine	Mania/mixed features, depression	5HT_2c_ antagonist 5HT_2a_ antagonist 5HT_7_ antagonist 5HT_2b_ antagonist 5HT_6_ antagonist 5HT_7_ α_2B_ antagonist D_3_ antagonist H_1_ antagonist α_1_ antagonist α_2A_ antagonist α_2C_ antagonist D_2_ antagonist D_1_ antagonist 5 HT_5a_ antagonist 5 HT_1a_ partial agonist H_2_ antagonist α_1_ antagonist	Sedation, EPSEs, postural hypotension
Cariprazine	Mania/mixed features	D_3_ partial agonist D_2_ partial agonist 5HT_2B_ antagonist 5HT_1A_ partial agonist 5HT_2A_ antagonist H_1_ antagonist 5HT_7_ antagonist 5HT_2C_ antagonist α _1A_ antagonist	Akathisia, EPSEs, nausea
Lurasidone	Mania/mixed features, depression	5HT_7_ antagonist D_2_ antagonist 5HT_2A_antagonist 5HT_1A_antagonist α_2_ antagonist	EPSEs, sedation
Olanzapine	Mania/mixed features, depression^c^	H_1_ antagonist 5HT_2A_ antagonist 5HT_2C_ inverse agonist D_2_ antagonist M_1_-M_5_ antagonist	Sedation, weight gain, dyslipidaemia
Quetiapine	Mania/mixed features, depression	H_1_ antagonist α_1A_ antagonist α_2C_ antagonist α_1B_ antagonist α_2B_ antagonist 5HT_2A_ antagonist D_2_ antagonist 5HT_7_ antagonist Norquetiapine (metabolite of quetiapine) H_1_ antagonist 5HT_2B_ antagonist M_3_ antagonist M_5_ antagonist M_1_ antagonist 5HT_1A_ partial agonist 5HT_2A_ antagonist NET inhibitor α_1_ antagonist D_2_ antagonist (higher doses)	Sedation, weight gain, postural hypotension
Risperidone	Mania	5HT_2A_ inverse agonist D_2_ antagonist 5HT_7_ antagonist α_1_ antagonist 5HT_2C_ inverse agonist H_1_ antagonist α_2_ antagonist	EPSEs, weight gain, elevated prolactin, postural hypotension

^a^Based on RCT and meta-analytic evidence in text, above

^b^Adapted from Maudsley Psychosis Prescribing Guidelines

^c^Effective in depression in combination with fluoxetrine

Discontinuation rates as adjunct varied from a hazard ratio of 0.66 (ziprasidone) to 0.89 (aripiprazole), with weight gain (defined as increase of > 7%) noted when meta-analyzing all antipsychotics.

An antipsychotic not examined in this review was lurasidone, which has an FDA license as monotherapy and adjunctive treatment to lithium and divalproex for acute treatment of bipolar depression, and asenapine, which has recently been examined in a maintenance trial. Following a 6 week double blind placebo controlled RCT of lurasidone monotherapy or adjunctive treatment with lithium or divalproex, participants were randomised to extended 6-month trial of lurasidone as monotherapy or adjunct. Though not primary outcome, treatment‐emergent mania occurred in 1.3% in the monotherapy group, and in 3.8% in the adjunctive group. Among extension study baseline responders, 10.2% met post hoc criteria for depression relapse during 6 months of treatment in the monotherapy group, 10.2% meeting relapse criteria in the adjunctive therapy group. The nature of the trial makes it difficult to compare depression and mania relapse to other treatments, though the low incidence of manic relapse should be noted (Ketter et al. 2016). A recent 26 week double-blind placebo controlled trial of asenapine maintenance therapy in 253 people with bipolar disorder found a statistically significantly longer time to recurrence of any mood episode (manic or depression), HR = 0.16 for manic episode, HR = 0.35 for depressive episode, though not for mixed episodes (though the study may have been under-powered, these being post hoc analyses) (Szegedi et al. 2018).

To this literature should be added RCTs of long-acting injections (LAIs). A randomized placebo controlled 52 week trial of aripiprazole depot showed a beneficial effect in Bipolar 1 illness, with a hazard ratio of 0.45 in recurrence of any mood episode, seen predominantly with manic episodes, mirroring evidence for oral aripiprazole (Calabrese et al. 2017). Similar efficacy is also seen for risperidone LAI, versus placebo, from two trials, of 18 and 24 months’ duration (Quiroz et al. 2010; Vieta et al. 2012), in relapse prevention, with combined risk ratio of 0.42 for manic, hypomanic or mixed symptoms, though not for depression relapse. A review summarising three trials of LAI versus oral antipsychotics found no difference in relapse rates, though sensitivity analysis showed benefit in people with rapid cycling illness (Kishi et al. 2016).

The first trial to compare the efficacy of antipsychotic monotherapy to lithium for relapse prevention in BD examined olanzapine versus lithium for relapse to any mood episode. This included open label co-treatment for 6–12 weeks, double blind taper over 4 weeks, and double-blind monotherapy for 48 weeks. It found no inferiority for olanzapine for the primary outcome, hospitalization for any mood episode (Tohen et al. 2005). A 2014 meta-analysis, by Miura et al., conducted pairwise and network meta-analyses examining efficacy and tolerability of treatments for the maintenance phase of BD. Network meta-analysis accounts for problems seen in conventional meta-analyses, where pairwise comparisons are made, and enables comparison amongst different interventions, given there is a common comparator within the network. 33 trials were included in the network analysis, examining 17 compounds/combinations. The antipsychotics examined were aripiprazole, olanzapine, paliperidone, quetiapine and risperidone LAI. It found all interventions were more effective than placebo in preventing relapse, apart from aripiprazole, carbamazepine, imipramine, and paliperidone. It should be noted that more recent studies of aripiprazole were not included. Only lithium and quetiapine were more effective than placebo for preventing depression relapse. The quality of evidence was variable, with the best evidence existing for lithium, in addition to other outcomes (e.g., social functioning). This led the authors to conclude that lithium should remain first choice therapy for maintenance treatment (Miura et al. 2014). What is striking from this meta-analysis of maintenance treatment is the degree of similarity of effect to that seen in acute treatment trials-a point made by Taylor, who found a close association between best estimates of effect versus placebo on relapse prevention against response rates for acute episodes reported in other network meta-analyses (correlation for manic or mixed episodes, *r *= – 0.91, p = 0.01) and a similar trend for depressive episodes (*r *= – 0.79, p = 0.06) (Taylor 2014). This suggests a true effect, as opposed to statistical artefact, and also suggests treatments that are effective acutely may be effective for prophylaxis. This was recently borne out by a meta-analysis by the same author, showing lithium had acute effects on relapse to mania, within 4 weeks (Taylor 2018).

Given the existing evidence for lamotrigine, licensed in the USA and EU for prevention of depression relapse in people with Bipolar 1 illness with predominantly depressive episodes (Goodwin et al. 2004), it is worth comparing its effectiveness to antipsychotics-particularly for BD after a depressive episode. The Miura network meta-analysis (above) (Miura et al. 2014) gave an effect size for lamotrigine of 0.69 (95% CI 0.5–0.94) for depressive episode, relapse or recurrence. Comparative values for antipsychotics were olanzapine 0.8 (95% CI 0.57–1.12), quetiapine 0.48 (95% CI 0.34–0.67), risperidone LAI 1.32 (95% CI 0.84–2.09), and 0.76 (95% CI 0.61–0.93) for lithium. At a pragmatic level, lamotrigine is commonly added to other maintenance treatment, including antipsychotics, and an important contribution to the literature is the CEQUEL trial (Geddes et al. 2016). This double-blind placebo-controlled RCT enrolled people with a new depressive episode to lamotrigine or placebo, finding reduction in depression symptoms (measured by the Quick Inventory of Depressive symptomatology-self report (QIDS-SR) at 12 weeks and 52 weeks (the latter being statistically significant, − 2.69, 95% CI − 4.89 to − 0.49]; p = 0.017), with greater improvement in those not receiving folic acid.

The only maintenance trial of antipsychotics in post-first episode mania recruited 61 people with first episode manic psychosis whose symptoms had responded to quetiapine and lithium and randomized them to quetiapine (mean dose 437.5 mg) or lithium (mean level 0.6 mM). Using a mixed-model, repeated measures design they found lithium superior to quetiapine on clinical measures (Young Mania Rating Scale (YMRS), Brief Psychiatric Rating Scale (BPRS), clinical global impression), and functioning (Berk et al. 2017; Geddes et al. 2016).

In an effort to combine knowledge about drug efficacy in maintenance treatment with the clinical picture of BD, Popovic et al. (2012) analyzed trials of greater than 24 weeks, assigning a polarity index, giving a numerical value for prophylactic effects versus depression (< 1) and mania (> 1). Examples include 4.38 for aripiprazole and 0.4 for lamotrigine. This is intuitively appealing, though-for compounds with equivalent efficacy (e.g. lithium, 1.39 and quetiapine, 1.14), interpretation may be more difficult.

For effectiveness, a recent national (Finnish) register study examined rehospitalization of people with bipolar disorder, for a mean of around 7 years. It found risperidone LAI (HR, 0.58 [95% CI 0.34–1.00]), gabapentin (HR, 0.58 [95% CI 0.44–0.77]), perphenazine long-acting injection (HR, 0.60 [95% CI 0.41–0.88]) and lithium carbonate (HR, 0.67 [95% CI 0.60–0.73]) were associated with lowest risk of psychiatric rehospitalization. The authors acknowledged possible confounders, though adjusted for individual variability by using each patient as their own control (Lähteenvuo et al. 2018; Popovic et al. 2012; Berk et al. 2017). The suggested use of LAIs is interesting, given the degree of non-adherence to medication seen in BD (20–66%) (Lingam and Scott 2002), though the literature from schizophrenia RCTs shows no difference in relapse rates compared to oral medication (Kishimoto et al. 2014).

The superiority of lithium has been echoed in a naturalistic study using a UK primary care database of around 500 people with BD, where the authors examined use of olanzapine, quetiapine, lithium, and sodium valproate monotherapy. They examined treatment failure, defined as addition of other psychotropic medication or discontinuation, and found longest time to failure for lithium, 2.05 years (95% CI 1.63–2.51), compared to 0.76 years (95% CI 0.64–0.84) for quetiapine, 0.98 years (95% CI 0.84–1.18) valproate, and 1.13 years olanzapine (95% CI 1.00–1.31) (Hayes et al. 2016). The authors were able to control for a number of confounding factors, though unable to allow for the effects of manic or depressive episodes.

## Adverse effects of antipsychotics

The main adverse events noted with second generation antipsychotics include metabolic effects (including weight gain, effects on glucose metabolism, cholesterol and prolactin), sedation, sexual dysfunction, as well as extrapyramidal symptoms (though to a lesser degree than FGAs) (Young et al. 2015). When considering adverse events, it should be noted that effects are heterogenous between compounds (Leucht et al. 2013), and that interventions are available to ameliorate side-effects, such as weight gain (Young et al. 2015; Maayan et al. 2010). An analysis using the same UK dataset described above found increased rates of hypertension, and 15% weight gain, compared to lithium (valproate HR 1.62; 95% CI 1.31–2.01; p < 0.001, olanzapine HR 1.84; 95% CI 1.47–2.30; p < 0.001, quetiapine HR 1.67; 95% CI 1.24–2.20; p < 0.001).

## Methodological issues in appraising the evidence

When appraising RCTs of maintenance treatments in BD, it should be acknowledged that follow-up rates are low-as low as 10% in some cases, with people excluded from trials after experiencing worsening of symptoms, such as recurrence of a mood episode. This limits interpretation of results, given that natural history of illness may require prolonged treatment with prophylactic medication before beneficial effects may be seen. A related issue is the detection of effects on depression episodes-inclusion criteria for trials is an initial episode of mania and given that initial polarity may predict future episode polarity, the ability to detect effects on depression may be affected, as a result of inadequate statistical power (though quetiapine’s effects on this outcome should be noted). As above, most trials of antipsychotics adopt an enriched design, where participants are stabilized on the antipsychotic before randomisation, i.e. preferentially choosing responders-though this probably reflects clinical practice. It is worth bearing in mind that those taking part in these studies have generally had poor responses to lithium or valproate (Lindström et al. 2017). Observational studies are notoriously difficult to interpret, given the effects of confounding factors, and selection bias. It may therefore be helpful to examine aetiological mechanisms and the biology of treatment response.

## The mechanism of action of treatment response in bipolar disorder

From the above evidence it is clear that response to treatment is heterogenous and that lithium, and to a lesser extent, quetiapine, appear most likely to have efficacy in preventing relapse to depression. Lithium also appears to have anti-suicidal effects. An understanding of the pharmacology and neurobiology of treatment response enables one to unpick the difference between lithium and specific antipsychotics in maintenance therapy.

Various hypotheses exist for lithium response and can be grouped into those regulating cell membrane properties, cell membrane transport and ion distribution, neurotransmitter regulation, and intracellular signalling, linked at various levels. An example is its effects on Glycogen Synthase Kinase (GSK) 3β inhibition, with phosphorylation of GSKβ through a Ca^2+^channel-mediated pathway, with inhibition of AKT phosphorylation and multiple downstream signalling effects (Alda 2015). Similarly, effects on the dopamine system occur downstream of dopamine D_2_ receptors (Ashok et al. 2017).

Second-generation antipsychotics (“atypical” antipsychotics”) are heterogenous, and this classification is based on the pharmacology of clozapine, the first antipsychotic found not to cause catalepsy in rodents, and clinically significant movement disorder (Gründer et al. 2009). They generally have less affinity for the D_2_ receptor than first generation antipsychotics, such as haloperidol, and more affinity for receptor sites such as the 5HT_2A_ receptor (an exception being amisulpride), though there is little evidence of an association between this and effectiveness. When examining the antipsychotics, it is worth considering the Neuroscience-based nomenclature (NbN) which classifies psychotropic medication on the basis of their pharmacological profiles (Zohar et al. 2014). For those antipsychotics approved for maintenance treatment in BD this is as follows; aripiprazole (receptor partial agonist (D_2_, 5HT_1A_), receptor antagonist (5HT_2A_)), olanzapine (receptor antagonist D_2,_ 5HT_2_), quetiapine (immediate-release) (receptor antagonist D_2_, 5HT_2_), reuptake inhibitor (norepinephrine transporter), ziprasidone (receptor antagonist D_2_, 5HT_2_). Given their effects in acute bipolar depression it is also worth noting the properties of asenapine (receptor antagonist 5HT_2_, D_2_, norepinephrine, alpha 2) and lurasidone (receptor antagonist D_2_, 5HT_2_).

This nomenclature, with a focus on receptor affinity and selectivity give some clue as to the pharmacological mode of action necessary for preventing mania- and to a lesser extent, depression. In terms of neurobiological research, in mania, there is a paucity of in vivo studies, with one Positron Emission Tomography (PET) study suggesting no elevation in dopamine synthesis capacity in mania, compared to controls (Yatham et al. 2002), though an elevation in mania with psychotic symptoms (Jauhar et al. 2017). However, taking into account the clinical data, pharmacological studies in healthy volunteers and post-mortem studies, it does appear that dopamine antagonism has a role in prophylaxis of mania, though lithium’s downstream effects on the dopamine signalling is difficult to reconcile with this (Ashok et al. 2017). The lack of effects of dopamine antagonists in depression suggests a non hyper-dopaminergic mechanism, with no clear dopaminergic basis for quetiapine and possibly asenapine’s effects on preventing depression (effects on other neurotransmitters such as serotonin (e.g. 5HT_7_ receptors being a possibility). Other possible mechanisms include effects on the NMDA system (explaining benefits of ketamine in bipolar depression (Bauer et al. 2018), and possible hypo-dopaminergia explaining the reported positive results of phase III trials of cariprazine in bipolar depression (https://www.allergan.com/news/news/thomson-reuters/allergan-announces-fda-acceptance-of-supplemental).

## What do guidelines suggest?

Most National guidelines continue to support use of lithium as first-line therapy in the maintenance treatment of bipolar disorder [e.g., *NICE* (https://www.nice.org.uk/guidance/cg185/chapter/key-priorities-for-implementation); British Association of Psychopharmacology (Goodwin et al. 2016)], with specific antipsychotics recommended for acute treatment. The use of prophylactic agents (grouping together conventional mood stabilizers and antipsychotics) is considered on an individual basis, based on efficacy and tolerability, with no explicit guidance given by the World Federation of Societies of Biological Psychiatry (WFSBP) (Grunze et al. 2013) or the Roy al Australian and New Zealand College of Psychiatrists guidelines (Malhi et al. 2015). The International College of Neuropsychopharmacology (CINP) guidelines (Fountoulakis et al. 2017) have specific suggestions for the clinical picture, with quetiapine or olanzapine monotherapy suggested if predominantly depressive polarity or no predominant polarity, and lithium, aripiprazole, olanzapine, paliperidone, quetiapine, risperidone (including RLAI) for predominant manic polarity, and olanzapine or aripiprazole plus a mood stabilizer for frequent mixed episodes and lithium for rapid cycling illness. The Canadian Network for Mood and Anxiety Treatment (CANMAT) and International Society for Bipolar Disorders (ISBD) 2018 guidelines for the management of patients with bipolar disorder are also more inclusive of other agents, suggesting continuation of treatments used in the acute episode. First-line suggestions include lithium, quetiapine, divalproex, and lamotrigine monotherapy. To these are added asenapine and aripiprazole (oral and LAI). They also suggest combination quetiapine with lithium/divalproex first-line. An important distinction that these guidelines make is for the use of olanzapine as second-line therapy, on account of its metabolic effects, as well as pointg out use of some SGAs adjunctively for short periods of time (Yatham et al. 2018).

## The controversial case of treatment of first episode mania

A good example of the issues regarding use of antipsychotics in maintenance treatment in BD comes from the clinical care of people presenting with first episode mania, within first episode psychosis (FEP) services (Jauhar et al. 2019). First-line pharmacotherapy will almost always be with SGAs, which will be continued for at least a year to 18 months, and then either continued or discontinued. Treatment with SGAs in this cohort is associated with more prominent metabolic effects and weight gain, which will have effects on treatment adherence, which can be poor (Whale et al. 2016). This is the biggest factor predicting relapse (Zipursky et al. 2014), with relapse of mania in FEP having more significant consequences in terms of hospitalizations than other psychotic illnesses such as schizophrenia (Chang et al. 2016). To date, there is little data on use of lithium or other mood stabilizers in this population over the longer-term, and whether this has an effect on treatment adherence or illness course.

## Pragmatic clinical questions

### Which patients may benefit from antipsychotic monotherapy?

Given that lithium is the gold standard, and that antipsychotics have differential effects at various poles of illness, when should antipsychotic monotherapy be considered? This would pertain to people who are unable to tolerate, do not have a good response or do not wish to take lithium. Monotherapy with a LAIs could be considered for those who have problems with adherence.

#### Use of combination antipsychotics

The use of combination antipsychotics is generally discouraged, on account of increased side-effect burden, and lack of clear efficacy-even in augmentation of clozapine for people with treatment-resistant psychoses. One area where antipsychotic combination could be considered is when hyperprolactinaemia occurs with an antipsychotic, and this cannot be changed, for reasons of efficacy/other side-effects. In these cases use of adjunctive aripiprazole is justified, in keeping with trial evidence (Raghuthaman et al. 2015) and prescribing guidelines (Taylor et al. 2018).

### Optimal duration of maintenance antipsychotic treatment

There is little evidence to guide decision-making, regarding length of antipsychotic maintenance treatment. In cases of combination therapy (e.g. with lithium) this would depend on the initial use of antipsychotic, though use as monotherapy would be equivalent to the guidance given for lithium, in terms of length of treatment, with appropriate monitoring of side-effects.

## Conclusions and future directions

Second generation antipsychotics are increasingly used for maintenance treatment in bipolar disorder, as monotherapy or adjunctive therapy. Although lumped together as one class, their pharmacological properties, efficacy and tolerability in bipolar disorder are varied. This is particularly the case for prevention of depressive episodes, where their clinical effects appear less pronounced (with the exception of quetiapine, and possibly asenapine and lurasidone). For people who are intolerant or unable to adhere to lithium use, maintenance treatment with antipsychotics seems reasonable, with individualized care regarding side effects and their management-and possible use of other agents for prevention of depression (e.g. ketamine), in conjunction with newer digital technologies, for early relapse signs and monitoring (Bauer et al. 2018). Future studies examining the neurobiology of treatment response in bipolar depression, as well as longer-term trials with various compounds, focusing on treatments acting on differing poles of illness are required.
